# Comparative study of pathogenic and non-pathogenic *Escherichia coli* outer membrane vesicles and prediction of host-interactions with TLR signaling pathways

**DOI:** 10.1186/s13104-018-3648-3

**Published:** 2018-08-01

**Authors:** Ava Behrouzi, Farzam Vaziri, Farhad Riazi Rad, Amir Amanzadeh, Abolfazl Fateh, Arfa Moshiri, Shohreh Khatami, Seyed Davar Siadat

**Affiliations:** 1 Department of Mycobacteriology and Pulmonary Research, Pasteur Institute of Iran, Tehran, Iran; 2 Microbiology Research Center (MRC), Pasteur Institute of Iran, Tehran, Iran; 3 Department of Immunology, Pasteur Institute of Iran, Tehran, Iran; 4 National Cell Bank of Iran, Pasteur Institute of Iran, Tehran, Iran; 5 Department of Biochemistry of Iran, Pasteur Institute of Iran, Tehran, Iran

**Keywords:** Outer membrane vesicles, Toll like receptor, *Escherichia coli*, Signaling

## Abstract

**Objective:**

The intestine is the major defensive barrier in the body by having more than 60% of the immune cells in the intestinal mucosa. The aim of this study was to evaluate the Toll like receptor (TLR) signaling pathways and immune response profiles, against outer membrane vesicles (OMVs) in pathogenic and non-pathogenic strains of *Escherichia coli*.

**Results:**

Our results demonstrated that despite inducing inflammatory and regulatory responses to OMVs released by both strains, there is a remarkable difference in the nature and severity of these responses between the two strains. Following the production and release of OMV by the pathogenic strain, the expressions of the pro-inflammatory cytokines were significantly elevated, in comparison to the non-pathogenic strains. Eventually, our findings suggest that OMV released by the pathogen strain might be colonized, causing inflammation, eliminating the tight junctions of epithelial cells and damaging underlying cells, without the presence of IL-17 at the inflammation site. This could have happened to prevent the development of more severe inflammation, which could lead to the inhibition of colonization. The production of IL-10 is also preventing such inflammations. On the other hand, OMV released by non-pathogenic *E. coli* appears to influence intestinal homeostasis by causing more anti-inflammatory responses and mild inflammation.

**Electronic supplementary material:**

The online version of this article (10.1186/s13104-018-3648-3) contains supplementary material, which is available to authorized users.

## Introduction

The human intestine is densely populated by approximately 10^14^ various bacteria, including the commensal ones. Hence, it is known as an organ with the densest and most diverse microbial population [1]. Moreover, the intestinal tract is the largest immune barrier [1, 2]. There are over 60% of all immune cells in the intestinal mucosa, capable of identifying and managing potential invasive agents, while contributing to the inhibition of unregulated responses [3, 4]. The outer membrane vesicles (OMV) have double-layered spherical structures with 20–250 nm diameters, produced and secreted by many bacteria. These vesicles are used for secretion of many secretory proteins and other active compounds, and can help to establish cellular communications without direct contact with the cells [5, 6]. The crosstalk between microbiota and the infected intestinal cells are the proteins or secretory factors, such as OMV [7, 8]. Considering the presence of non-pathogenic *Escherichia coli* as the first intestinal bacteria, established in the early life and the significance of OMV in pathogenesis, as well as the importance of Toll like receptors (TLR) in the intestinal epithelial cells [9], here we aimed to compare the signaling pathways and cytokine responses, activated by the presence of OMV extracted from the pathogenic and non-pathogenic *E. coli* strains, in Caco2 epithelial cell line.

## Main text

### Methods

#### Preparation of outer membrane vesicles

OMVs were extracted from pathogenic and non-pathogenic strains of *E. coli*, as previously reported [10].

#### Electron microscopy

Electron microscopy was used to verify the stability and maintenance of the structures and shapes of the OMV, during the purification steps. For this purpose, after transferring the OMV onto formvar/carbon coated nickel grids, washing with PBS, containing 0.5% BSA and 0.1% gelatin, and fixing with 1% glutaraldehyde, the grids were subjected to negative staining with potassium phosphotungstate, and observed by a Field Emission scanning electron microscope (FE-SEM) (HITACHI CS-4160).

#### Cell line and culture conditions

Caco2 human colon adenocarcinoma cell line was obtained from the Cell Bank of Pasteur Institute of Iran. The cells were cultured routinely at 37 °C, in the presence of 5% CO_2_ in Dulbeccos Modified Eagle Medium (DMEM) (Gibco, Carlsbad, CA, USA.), supplemented with 25 mM HEPES (Gibco), 10% FBS (Gibco), Penicillin G (100 u/ml) (Gibco), and Streptomycin (100 μg/ml) (Gibco).

#### Inoculation of OMV

Inoculations were performed on two groups of cells, one consisted of Caco2 cells. Both groups were inoculated with 50 μg/ml of the extracted OMV. The first group was incubated for 24 h for real-time PCR. The second group was incubated for 72 h. The cell supernatants were then collected and finally stored at − 80 °C for the cytokine assays.

#### RNA extraction, cDNA synthesis and real time PCR

Total RNA was extracted from the Caco2 cells, using the High Pure RNA Isolation Kit (Qiagen, Hilden, Germany), according to the manufacturer’s instructions. One microgram of the extracted RNA was applied to reverse transcription, using the RT First Strand Kit (Qiagen, Hilden, Germany). SYBR-Green-based real-time PCR was performed, using a Toll-like receptor Signaling Pathway RT^2^ Profiler PCR Array kit (Qiagen, Hilden, Germany), according to the manufacturer’s instructions.

#### Cytokine assay in cell supernatants

The supernatants of Caco2 cells, incubated with OMVs for 72 h, in the presence of specific concentrations of OMV, and stored at − 80 °C, used to quantitate the secreted IL-10, IL-4 and IL-17, using the Human ELISA kits (Mabtech, Sweden), according to the manufacturer’s instructions. All specimens were tested as duplicates.

#### Statistical analyses

Statistical analyses were performed on real-time PCR data, using the Qiagen online analysis system, according to the manufacturer’s proposed guidelines, as well as PathVisio software, KEGG String, and WikiPathways online websites (https://www.wikipathways.org/index.php/WikiPathways). The statistical analyses for the cytokine assays were performed by GraphPad Prism 6 software (GraphPad Software Inc., USA), using independent *t* test and ANOVA; p values < 0.05 were reported as statistically significant.

### Results

#### Vesicle sizes, protein migration and LPS contents of the extracted OMV

**Fig. 1:**
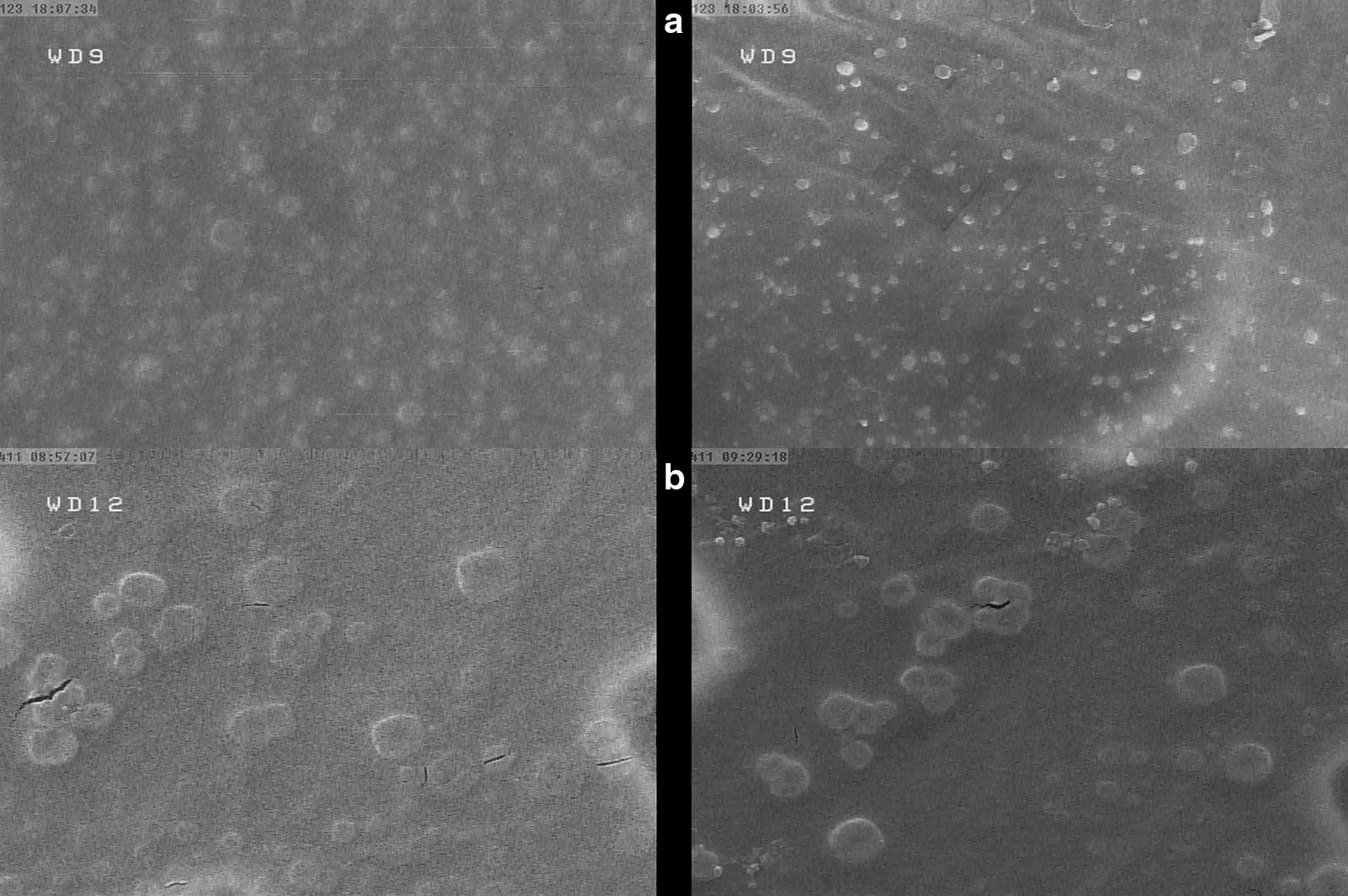
SEM image of the outer membrane vesicles, indicating image (**a**) for the pathogenic bacterial outer membrane vesicles produced at a smaller size of 20–70 nm in large amount, and image (**b**) for the non-pathogenic bacterial outer membrane vesicles produced at a larger size of 45–270 nm in lower amount

The extracted OMV from the pathogenic strain were 20–75 nm in size, while the sizes of OMV from the non-pathogenic strain were 45–270 nm. As shown in Fig. 1, the pathogenic strain had a potential to produce much smaller vesicles than the non-pathogenic strain.

#### *Escherichia coli* OMVs leads to anti-inflammatory responses (ELISA Results)

**Fig. 2:**
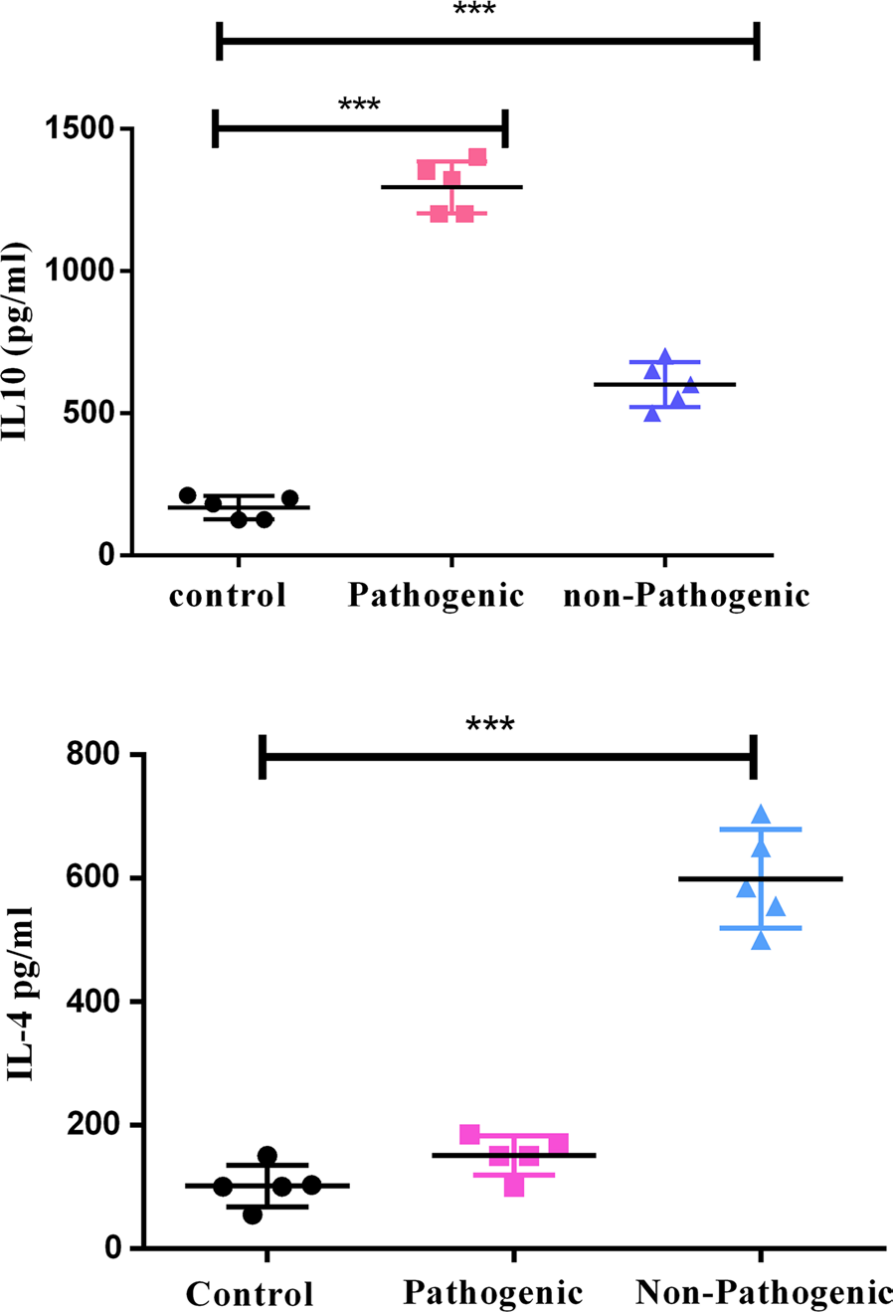
The outer membrane vesicles of pathogenic and non-pathogenic *E. coli* are able to induce the secretion of anti-inflammatory cytokine IL-10 and IL-4. The 72-h cellular soup collected from the Caco2 cell line was measured at a concentration of 50 μg OMV. *** indicates a significant increase in the case group compared to the control group (p value < 0.001)

OMVs released by pathogenic and commensal bacteria, have immunosuppressive activity in different cell types. The release of IL-10 in both strains was significantly higher than the control group (p value < 0.05). Both strains showed a significant increase in secretion of IL-10 levels. However, this difference was significantly higher for the pathogenic bacteria, compared to the non-pathogenic bacteria (Fig. 2). Compared with real time PCR results indicated that in the presence of Caco2 alone, increased IL-10 expressions were observed in both pathogenic and non-pathogenic strains (p < 0.05). The increased expression was 24 times higher in the pathogenic strain, while the non-pathogenic strain showed only a threefold higher expression.

The level of IL-4 secretion in the pathogenic strain showed no significant increase, compared to the control group; however, a significant increase in this cytokine was observed in the non-pathogenic strain, compared to the control group (p < 0.05) (Fig. 2). The level of IL-17 secretion was also measured in both strains. No secretion of this cytokine was observed in the non-pathogenic strain. However, an increase in the level of IL-17 was seen in the pathogenic strain, although this increase was not significant, compared to the control group (data not shown).

#### The OMVs of pathogenic and non-pathogenic strains of *E. coli* have regulatory effects on the associated genes with TLR pathways (qPCR Results)

**Fig. 3:**
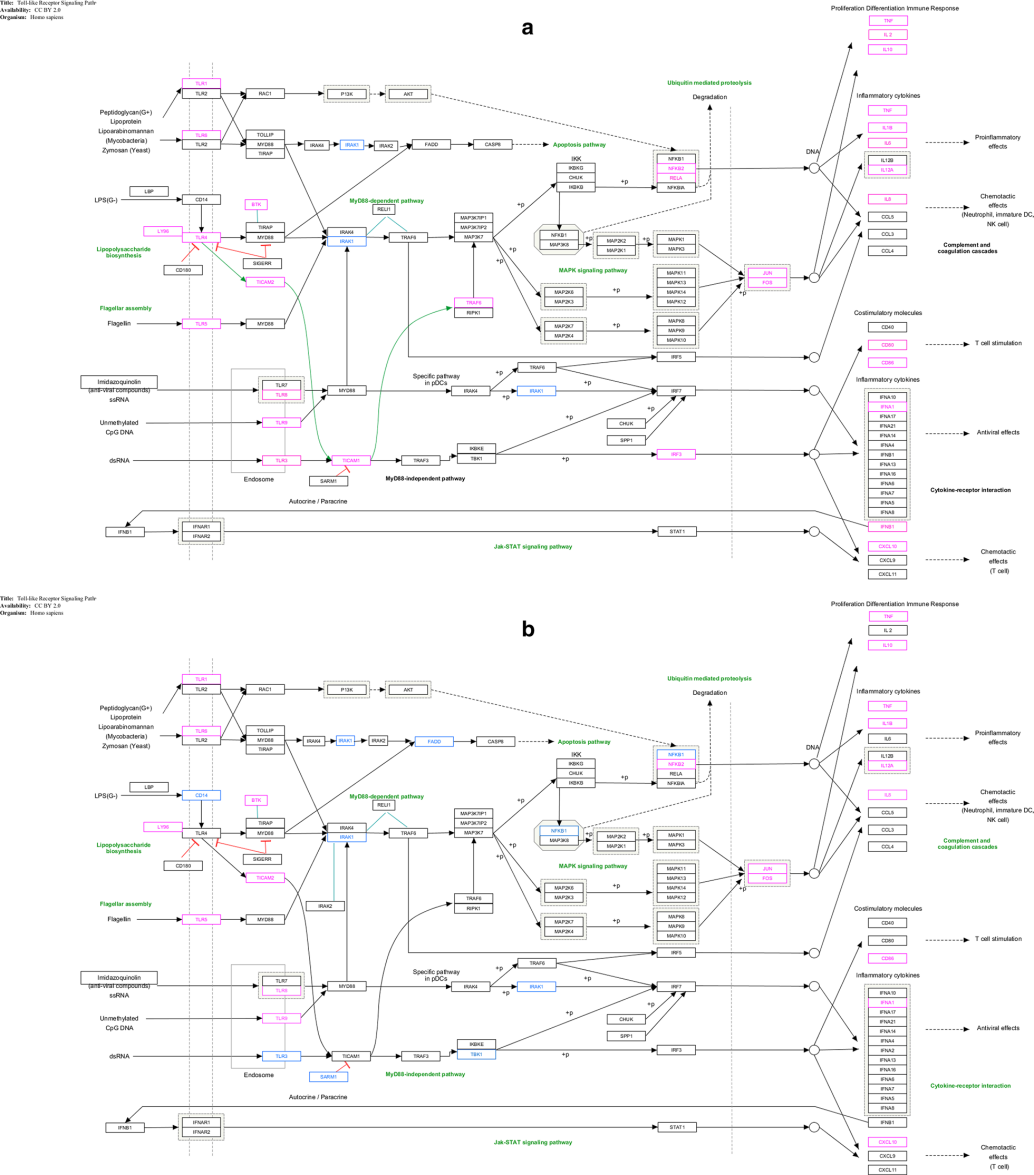
Analyzing Toll-like receptor signaling pathway by QIAGEN, KEGG and wikipathway websites and drawing pathway by Pathvisio software. The pathway involves up or down-regulating genes induced by pathogenic (**a**) and non-pathogenic (**b**) *E. coli* OMV, indicating red for the up-regulating genes and blue for the down-regulating genes

In this study, the expressions of most TLRs were increased. In the pathogenic strain, the expression of TLR 1-3-4-5-6-7-8 and TLR9 was increased, compared to the untreated cell. The highest levels of expression were observed in TLR7, TLR1 and TLR8, which were estimated to be 31, 27 and 22 times higher than untreated cell, respectively. In pathogenic strain, the expression of TLR 6, 9, 4, 5 and 3 showed 16, 13, 12, 10 and fivefold increase, respectively. However, the expression was increased in TLR 1-5-6-7-8 and TLR9 was increased in the non-pathogenic strain, but all showing twofold increase in expression; only TLR9 showed fourfold increase in the expression. In case of inflammatory and anti-inflammatory cytokines, the increased expressions of IL10-12A-1A-1B-2-6 and IL8 genes were also observed in the pathogenic strain, while IL10-12A-1A-1B and IL8 genes expression increased in the non-pathogenic strain. The highest level of expression in the pathogenic strain was observed for IL-10, IL-2, TNF and IL-6 with 24, 20, 15 and 12-fold increase, respectively. The expression levels were increased up to fivefold, in cases of IL-12A and IL1B, sevenfold in IL-8 and threefold in IL-1A. Meanwhile, IFNA1 and IFNB1 antiviral cytokines indicated 23- and 25-fold increases in the pathogenic strain, respectively. The non-pathogenic strain indicated threefold increase in IL-1B and IL-8 genes expression and the rest shows twofold increase (p < 0.05) (Fig. 3—Additional file 1). Clustergram plot (Additional file 2) by mean log twofold change of each of the genes in the Caco2 cell line, exposed to OMV of each pathogen and non-pathogenic bacteria, was mapped, using Qiagen’s online software.

### Discussion

OMV can induce inflammation in a wide range of human tissues. Recent studies have shown that OMVs released by commensal bacteria plays a major role in immune maturation, while OMV released by pathogenic bacteria facilitates infection and inflammation in the host. Our findings showed that despite the induction of inflammatory and regulatory responses, the nature and severity of these responses, with respect to the origin of OMVs, have significant differences. OMV are produced by both pathogenic and commensal bacteria [11] and suggested providing advantages for their parent bacteria [12, 13].

Our electron microscopy images demonstrated that the pathogenic *E. coli* produced more OMV than the non-pathogenic strains, consistent with results that have indicated tenfold OMV overproduction in other pathogenic *E. coli* and 25-fold overproduction of OMV in *Aggregatibacter actinomycetemcomitans* [14–16]. The size of the OMV could influence its penetration, so that OMV with a size smaller than 120 nm can enter the cell [14, 17]. OMV with smaller sizes are associated with better penetration and involvement of the Pattern Recognition Receptors (PRRs) within the cell [18, 19].

The OMV released by the pathogenic strain induced a high expression of pro-inflammatory and antiviral cytokines in the cell line; however, the OMV released by the non-pathogenic strain only led to a slight increase in the pro-inflammatory cytokines (IL-1β, IL-8 and TNF- α) productions. The assessment of OMV released by commensal *E. coli* has been shown to induce a mild inflammatory response in Caco-2 cell line [20–23]. Bacteria can use OMV to transfer their RNA to their host cells. In addition, bacterial RNA, penetrated into the eukaryotic cell may be detected by TLR3, 7, and 8 [18, 24, 25]. Hence, increasing the expression of TLRs, such as TLR3-7 and TLR8 is in agreement with our results. Here, IL-17, in the presence of Caco2 and OMVs showed no significant increase by any of the two strains, compared to the control group, which could be due to the increase in the expression of type I interferon genes and inhibition of IL-17. In fact, our hypothesis was based on the data that OMV released by the pathogens carries virulence factors that weaken the inflammatory responses by generating predominant responses toward type I interferon.

The TLRs play important roles in inducing pro-inflammatory cytokines, as well as participating in the induction of antiviral cytokines. The overexpression of pro-inflammatory cytokines (IL-1, IL-6, IL-8, IL-12 and TNF) by the pathogenic strain, compared to slight increases in IL-1, IL-8 and TNF expressions by the non-pathogenic strain, appears to be associated with overexpression of TLR due to OMV released by the pathogen. It has been shown that the OMVs from commensal *E. coli* strains do not increase the expression of cytokine genes and TLRs, associated with a mild inflammation [20].

One of the main bacterial virulence factors is LPS that is present as part of the OMV structure. LPS also plays an important role in the OMV biogenesis [26]. Our study showed that there are different levels of LPS in the structure of OMV, as indicated by approximately four times higher amounts of LPS in OMV of the pathogenic strain, compared to the non-pathogenic strain. Among the TLRs, TLR4 together with CD14 and LY96 (MD2) co-receptors collaborate in identifying and responding to LPS [27]. Studies have shown that lipid A in the OMV released by the pathogens, changes in comparison to the non-pathogenic strains [28]. A study by Soult and colleagues showed that OMV caused by both strains of pathogenic and non-pathogenic *E. coli* has the potential to increase the expression of adhesion protein, such as ICAM and E-Selectin proteins, and to increase the expression of IL-6 in the HUVECs. On the other hand, they stated that the NFkB nuclear translocation in HUVECs was increased in the presence of both strains, and since NFkB translocation was triggered by the activation of TLR-4, this fact may have been corresponding to the LPS in the OMV structure [29]. In another study, Soderblom et al. [30] on the T24 cell line showed that despite the production of pro-inflammatory responses by OMV, mediated by the LPS-TLR4-signaling pathway, in the absence of the TLR4 pathway, OMV may have the potential for producing an anti-inflammatory cytokine, such as IL-8, due to the presence of ompW, confirming the possibility of inducing pre-inflammatory responses in a non-TLR4 pathway. Research indicates that TLR4 may be activated in two MYD88-dependent and independent pathways and co-receptor CD14 is only important for the MYD88-dependent pathway. It seems that due to the reduction of CD14 expression in the non-pathogenic strain and a low concentration of LPS in the OMV structure, stimulation and activation of TLR4 pathway have been performed by a MYD88-independent pathway [27, 28, 31]. In the pathogenic strain, due to the high concentrations of LPS, measured in OMV, the lack of alteration of CD14 expression would not interfere with the MYD88-dependent pathway. However, regarding the reduced expression of IRAK1 in the MYD88-dependent pathway [32–35], it seems that the activation has been made through the MYD88-independent pathway in the pathogenic strain. In the present study, Caco-2 cells incubated with OMV released by the pathogenic strain showed high expression levels of IL-1a and IL-1b genes, compared to OMV released by the non-pathogenic strains. Furthermore, the comparison of OMV proliferation in the two strains showed the death of cells, due to OMV released by the pathogen.

In this study, we obtained the same result by Caco-2 cell line and observed an increase in the expression level of IL-10. The TLR4 signaling is important for maintaining the production of IL-10 by intestinal epithelial cells, which is a major mechanism for maintaining intestinal homeostasis, mediated by host-bacterial interactions [36]. Finally, our results suggested that OMVs released by the pathogenic bacterial strain lead to the development of inflammation, by inducing the expression of pro-inflammatory cytokines through the involvement of various TLRs by PAMPs on the luminal surface, so that they can use the inflammatory condition in order to obtain nutrients [37].

At the same time, OMV released by the pathogenic strain appeared to be capable of inducing anti-inflammatory cytokine IL-10, by engaging the TLR4 to be able to prevent the inflammation. However, OMV released by the non-pathogenic strain was unable to involve the TLRs and caused a low stimulation of TLR signaling compared to the OMV originated from the pathogenic strain; hence, it developed only a mild inflammation that possibly could maintain the intestinal homeostasis.

## Limitation

It also advocates for further studies to assess the interaction of another helpful microbiota with TLR signaling pathways in intestinal.

## Additional files

**Additional file 1.** Expression analysis of TLRs and interleukins in Caco2 stimulation. Caco2 after 24 h stimulation with OMVs. Data are presented as fold-change compared to untreated control cells. Statistical differences were assessed by the *t*-test. ∗ *p* ≤ 0.05, versus control cells.

**Additional file 2.** Clustergram plot of genes involved in TLRs signalling pathways. In order to demonstrate a heat map dendrograms, showing the co-regulated genes, a clustergram for the entire dataset was mapped, using non-supervised hierarchical clustering.

## Abbreviations

PRRs: pattern recognition receptors
TLR: Toll-like receptors
MYD88: myeloid differentiation primary response 88
IRAK: interleukin receptor-associated kinase
OMV: outer membrane vesicles
LY96/MD2: lymphocyte antigen 96

## References

[1] Gill SR, Pop M, DeBoy RT, Eckburg PB, Turnbaugh PJ, Samuel BS (2006). Metagenomic analysis of the human distal gut microbiome. Science.

[2] Ley RE, Peterson DA, Gordon JI (2006). Ecological and evolutionary forces shaping microbial diversity in the human intestine. Cell.

[3] Hooper LV, Macpherson AJ (2010). Immune adaptations that maintain homeostasis with the intestinal microbiota. Nat Rev Immunol.

[4] Round JL, Mazmanian SK (2009). The gut microbiota shapes intestinal immune responses during health and disease. Nat Rev Immunol.

[5] Schwechheimer C, Kuehn MJ (2015). Outer-membrane vesicles from Gram-negative bacteria: biogenesis and functions. Nat Rev Microbiol.

[6] Huang W, Wang S, Yao Y, Xia Y, Yang X, Li K (2016). Employing *Escherichia coli*-derived outer membrane vesicles as an antigen delivery platform elicits protective immunity against *Acinetobacter baumannii* infection. Sci Rep.

[7] Park K-S, Choi K-H, Kim Y-S, Hong BS, Kim OY, Kim JH (2010). Outer membrane vesicles derived from *Escherichia coli* induce systemic inflammatory response syndrome. PLoS ONE.

[8] Mirlashari MR, Lyberg T (2003). Expression and involvement of Toll-like receptors (TLR)2, TLR4, and CD14 in monocyte TNF-alpha production induced by lipopolysaccharides from *Neisseria meningitidis*. Med Sci Monit..

[9] McBroom AJ, Kuehn MJ (2007). Release of outer membrane vesicles by Gram-negative bacteria is a novel envelope stress response. Mol Microbiol.

[10] Wai SN, Takade A, Amako K (1995). The release of outer membrane vesicles from the strains of enterotoxigenic *Escherichia coli*. Microbiol Immunol.

[11] Kadurugamuwa JL, Beveridge TJ (1995). Virulence factors are released from *Pseudomonas aeruginosa* in association with membrane vesicles during normal growth and exposure to gentamicin: a novel mechanism of enzyme secretion. J Bacteriol.

[12] Kaparakis-Liaskos M, Ferrero RL (2015). Immune modulation by bacterial outer membrane vesicles. Nat Rev Immunol.

[13] Lai CH, Listgarten MA, Hammond BF (1981). Comparative ultrastructure of leukotoxic and non-leukotoxic strains of *Actinobacillus actinomycetemcomitans*. J Periodontal Res.

[14] Cecil JD, O’Brien-Simpson NM, Lenzo JC, Holden JA, Chen Y-Y, Singleton W (2016). Differential responses of pattern recognition receptors to outer membrane vesicles of three periodontal pathogens. PLoS ONE.

[15] Horstman AL, Kuehn MJ (2002). Bacterial surface association of heat-labile enterotoxin through lipopolysaccharide after secretion via the general secretory pathway. J Biol Chem.

[16] Lee EY, Bang JY, Park GW, Choi DS, Kang JS, Kim HJ (2007). Global proteomic profiling of native outer membrane vesicles derived from *Escherichia coli*. Proteomics.

[17] Amano A, Takeuchi H, Furuta N (2010). Outer membrane vesicles function as offensive weapons in host–parasite interactions. Microbes Infect.

[18] Kaparakis M, Turnbull L, Carneiro L, Firth S, Coleman HA, Parkington HC (2010). Bacterial membrane vesicles deliver peptidoglycan to NOD1 in epithelial cells. Cell Microbiol.

[19] Patten DA, Hussein E, Davies SP, Humphreys PN, Collett A (2017). Commensal-derived OMVs elicit a mild proinflammatory response in intestinal epithelial cells. Microbiology.

[20] Noppert SJ, Fitzgerald KA, Hertzog PJ (2007). The role of type I interferons in TLR responses. Immunol Cell Biol.

[21] Abdullah Z, Schlee M, Roth S, Mraheil MA, Barchet W, Böttcher J (2012). RIG-I detects infection with live Listeria by sensing secreted bacterial nucleic acids. EMBO J.

[22] Kanneganti T-D, Özören N, Body-Malapel M, Amer A, Park J-H, Franchi L (2006). Bacterial RNA and small antiviral compounds activate caspase-1 through cryopyrin/Nalp3. Nature.

[23] Pfeiffer JK, Virgin HW (2016). Transkingdom control of viral infection and immunity in the mammalian intestine. Science.

[24] Love AC, Schwartz I, Petzke MM (2014). Borrelia burgdorferi RNA induces type I and type III interferons via TLR7 and contributes to the production of NF-κB-dependent cytokines. Infect Immun.

[25] Petzke MM, Iyer R, Love AC, Spieler Z, Brooks A, Schwartz I (2016). Borrelia burgdorferi induces a type I interferon response during early stages of disseminated infection in mice. BMC Microbiol.

[26] Kulp A, Kuehn MJ (2010). Biological functions and biogenesis of secreted bacterial outer membrane vesicles. Annu Rev Microbiol.

[27] Rajaiah R, Perkins DJ, Ireland DD, Vogel SN (2015). CD14 dependence of TLR4 endocytosis and TRIF signaling displays ligand specificity and is dissociable in endotoxin tolerance. Proc Natl Acad Sci USA.

[28] Steimle A, Autenrieth IB, Frick J-S (2016). Structure and function: lipid A modifications in commensals and pathogens. Int J Med Microbiol.

[29] Soult MC, Lonergan NE, Shah B, Kim W-K, Britt L, Sullivan CJ (2013). Outer membrane vesicles from pathogenic bacteria initiate an inflammatory response in human endothelial cells. J Surg Res.

[30] Söderblom T, Oxhamre C, Wai SN, Uhlén P, Aperia A, Uhlin BE (2005). Effects of the *Escherichia coli* toxin cytolysin A on mucosal immunostimulation via epithelial Ca^2+^ signalling and Toll-like receptor 4. Cell Microbiol.

[31] Peng J, Yuan Q, Lin B, Panneerselvam P, Wang X, Luan XL (2010). SARM inhibits both TRIF-and MyD88-mediated AP-1 activation. Eur J Immunol.

[32] Berglund M, Thomas J, Hörnquist E, Hultgren OH (2008). Toll-like receptor cross-hyporesponsiveness is functional in interleukin-1-receptor-associated kinase-1 (IRAK-1)-deficient macrophages: differential role played by IRAK-1 in regulation of tumour necrosis factor and interleukin-10 production. Scand J Immunol.

[33] Casson CN, Copenhaver AM, Zwack EE, Nguyen HT, Strowig T, Javdan B (2013). Caspase-11 activation in response to bacterial secretion systems that access the host cytosol. PLoS Pathog.

[34] Hagar JA, Powell DA, Aachoui Y, Ernst RK, Miao EA (2013). Cytoplasmic LPS activates caspase-11: implications in TLR4-independent endotoxic shock. Science.

[35] Vanaja SK, Russo AJ, Behl B, Banerjee I, Yankova M, Deshmukh SD (2016). Bacterial outer membrane vesicles mediate cytosolic localization of LPS and caspase-11 activation. Cell.

[36] Hyun J, Romero L, Riveron R, Flores C, Kanagavelu S, Chung KD (2015). Human intestinal epithelial cells express interleukin-10 through Toll-like receptor 4-mediated epithelial-macrophage crosstalk. J Innate Immun.

[37] Siegel SJ, Weiser JN (2015). Mechanisms of bacterial colonization of the respiratory tract. Annu Rev Microbiol.

