# Supplementary figures and images for: Comparative study of pathogenic and non-pathogenic Escherichia coli outer membrane vesicles and prediction of host-interactions with TLR signaling pathways
Source: BMC Res Notes. 2018 Aug 1;11:539. doi: 10.1186/s13104-018-3648-3 (PMC6071399; doi:10.1186/s13104-018-3648-3)

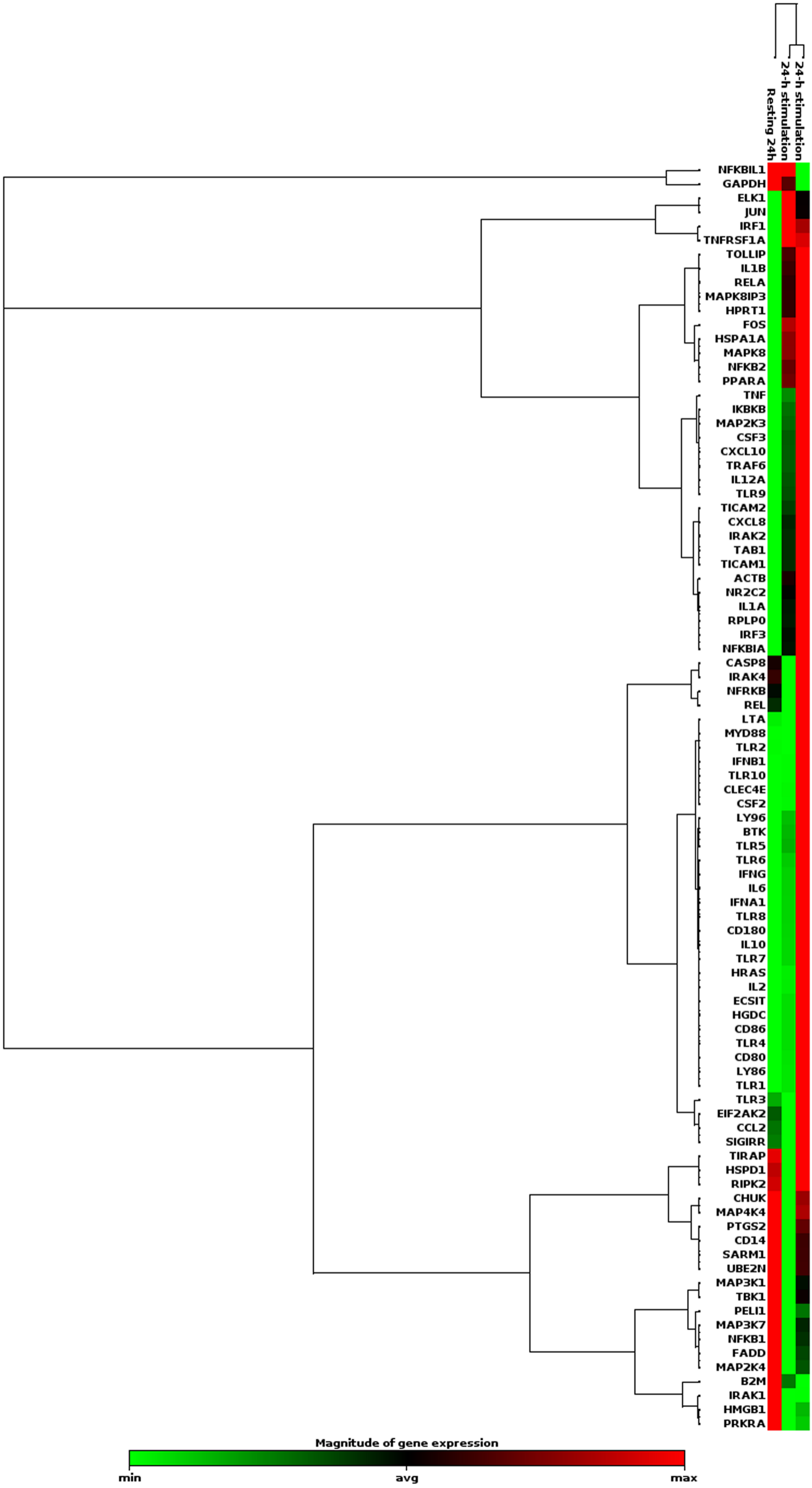

Supplement: Supplementary file 2 — Additional file 2. Clustergram plot of genes involved in TLRs signalling pathways. In order to demonstrate a heat map dendrograms, showing the co-regulated genes, a clustergram for the entire dataset was mapped, using non-supervised hierarchical clustering. [file 13104_2018_3648_MOESM2_ESM.pdf]
